# Incidental and Underreported Pleural Plaques at Chest CT: Do Not Miss Them—Asbestos Exposure Still Exists

**DOI:** 10.1155/2017/6797826

**Published:** 2017-06-05

**Authors:** Maria Antonietta Mazzei, Francesco Contorni, Francesco Gentili, Susanna Guerrini, Francesco Giuseppe Mazzei, Antonio Pinto, Nevada Cioffi Squitieri, Antonietta Gerardina Sisinni, Valentina Paolucci, Riccardo Romeo, Pietro Sartorelli, Luca Volterrani

**Affiliations:** ^1^Department of Medicine, Surgery and Neuroscience, Unit of Diagnostic Imaging, Azienda Ospedaliera Universitaria Senese, University of Siena, Siena, Italy; ^2^Italian College of Ethics and Forensic Radiology, Italian Society of Medical Radiology (SIRM), Milan, Italy; ^3^University of Siena, Siena, Italy; ^4^Unit of Diagnostic Imaging, Azienda Ospedaliera Universitaria Senese, University of Siena, Siena, Italy; ^5^Department of Radiology, Cardarelli Hospital, Naples, Italy; ^6^Unit of Occupational Medicine, Azienda Ospedaliera Universitaria Senese, University of Siena, Siena, Italy; ^7^Department of Medical Biotechnology, Unit of Occupational Medicine, Azienda Ospedaliera Universitaria Senese, University of Siena, Siena, Italy

## Abstract

Pleural plaques (PPs) may be a risk factor for mortality from lung cancer in asbestos-exposed workers and are considered to be a marker of exposure. Diagnosing PPs is also important because asbestos-exposed patients should be offered a health surveillance that is mandatory in many countries. On the other hand PPs are useful for compensation purposes. In this study we aimed to evaluate the prevalence, as incidental findings, and the underreporting rate of PPs in chest CT scans (CTs) performed in a cohort of patients (1512) who underwent chest CT with a slice thickness no more than 1.25 mm. PPs were found in 76 out of 1482 patients (5.1%); in 13 out of 76 (17,1%) CTs were performed because of clinical suspicion of asbestos exposure and 5 of them (38%) were underreported by radiologist. In the remaining 63 cases (82.9%) there was no clinical suspicion of asbestos exposure at the time of CTs (incidental findings) and in 38 of these 63 patients (60.3%) PPs were underreported. Reaching a correct diagnosis of PPs requires a good knowledge of normal locoregional anatomy and rigorous technical approach in chest CT execution. However the job history of the patient should always be kept in mind.

## 1. Introduction

Asbestos is a general term for a heterogeneous group of hydrated magnesium silicate minerals that have in common a tendency to separate into fibres [1]. It has long been used in roofing, insulators, brake pads, and gaskets, and in various workplaces and construction sites. Asbestos has been the largest single cause of occupational cancer in the United States and a significant cause of disease and disability from nonmalignant disease [2]. In Italy, the asbestos epidemic continues and is even increasing because of the country's industrial history. Up to the end of the 1980s, Italy was the second largest asbestos producer in Europe after the Soviet Union and the largest in the European Community, with a peak between 1976 and 1980 [3]. Furthermore asbestos imports to Italy reached a peak when they were already falling in the UK and US and the consumption curve of asbestos shows a lag time of about 10 years compared to many industrialized countries [4]. Asbestos fibres, inhaled and displaced by various means to lung tissue, may cause a spectrum of diseases including cancer (especially mesothelioma and lung cancer) and nonmalignant asbestos-related disease that refer to the following conditions: asbestosis, pleural thickening or asbestos-related pleural fibrosis (plaques or diffuse fibrosis), “benign” (nonmalignant) pleural effusion, and airflow obstruction [5, 6]. Pleural plaques (PPs) are usually asymptomatic and cause slight impairment of lung function only when they are extended in size [7–9]. However, they are the most common form of the pleuropulmonary abnormality consistent with asbestos exposure and are considered to be a marker of exposure, indicating an increased risk of pulmonary fibrosis or asbestos-related malignancies versus the general population [10]. In fact PPs may be a risk factor for mortality from lung cancer in asbestos-exposed workers, particularly in either smokers or former/ex-smokers [11]. Moreover the presence of PPs may help in considering asbestosis as a cause of interstitial lung disease predominating in the subpleural area of the lower lobes [12]. A recent Japanese study even found that in lung cancer patients the plaque extent had a significant positive relationship with the asbestos body concentration in lung tissue that represents a biomarker of past exposure [13]. However even if for certain types of asbestos the development of PPs is statistically correlated with malignant disease, the evidence is consistent with the hypothesis that PPs without other pleural disease are a marker of exposure, rather than an independent risk factor [14]. Diagnosing PPs is also important as asbestos-exposed patients should be offered a health surveillance that is mandatory in many countries. On the other hand PPs are useful for compensation purposes. In Italy PPs notification by physicians is required by law. From 2016 it is also mandatory to send the first medical certificate of PPs diagnosis to the Italian National Insurance Institute (INAIL). Doctors who fail to comply with these obligations may be fined. From a diagnostic point of view, in most screenings for pneumoconiosis, a chest radiograph is used as the standard method, but this procedure has important limitations in the detection of early subtle PPs, whereas a CT scan enables diagnosis of thin or tiny noncalcified plaques [10, 15–17]. Experienced CT readers can diagnose PPs with high confidence in most cases, which show the typical findings of bilateral, multiple, localised, pleural thickenings sparing the costophrenic angles. However, the CT features of PPs are sometimes equivocal in challenging cases and if the radiologists are not skilled in occupational diseases PPs could be underreported [18].

In this study we aimed to evaluate the prevalence, as incidental findings, and the underreporting rate of PPs in chest CT scans (CTs) performed in a cohort of patients who underwent chest CT with a slice thickness no more than 1.25 mm (high resolution protocol) at our department.

## 2. Material and Method

### 2.1. Study Design

This retrospective study was approved by the institutional review board of each participating centre, and the requirement for patient approval or informed consent for the retrospective analysis of anonymous images was waived. The study cases were identified by reviewing the radiological databases of the Diagnostic Imaging Unit at the Azienda Ospedaliera Universitaria Senese from January 2016 to June 2016. 1512 CTs fulfilled technical eligibility criteria (see CT scanning protocols section). All the CTs were independently reviewed by two radiologists in order to search for pleural thickening or asbestos-related pleural fibrosis (plaques or diffuse fibrosis); the presence of “benign” (nonmalignant) pleural effusion and asbestosis was also investigated.

### 2.2. Scanning Protocols

All CTs were performed using a 64-detector row CT scanner (Discovery 750 HD, GE Healthcare, Milwaukee, WI, USA). The field of view (FOV) of all eligible chest CTs had to include the rib cage. Since CT slice thickness varied according to the clinical indication, only the exams with a slice thickness no greater than 1.25 mm for pulmonary embolism detection or cancer evaluation (total number 960) and 1.25 mm for high resolution CT (HRCT, total number 552) were considered eligible for this study. In all patients chest CTs were performed without contrast medium administration; in oncological patients or patients with clinical suspicion of pulmonary embolism, a CT scan after administering contrast medium was also performed, and in the latter cases both scans (with and without contrast medium administration) were provided for review. Eligible HRCTs were acquired using a volumetric technique; in 273 out of 552 HRCTs, the scan was performed with the patient in a prone position because of the clinical suspicion of interstitial lung disease, to avoid possible parenchymal dysventilation in the dependent portions of the lung, mimicking lung fibrosis. Finally all CTs provided for review were reconstructed at window settings optimised for the assessment of the mediastinum.

### 2.3. Image Evaluation

Each CT examination, from which patient personal information has been removed, was analysed by two radiologists (with 16 and 8 years' experience in chest CT, resp., and 5 years of experience each as CT readers for asbestos-related thoracic diseases) who were blind to subjects' job history and possible history of asbestos exposure. The two readers independently assessed the pulmonary and pleural lesions as consistent with asbestos exposure and reached a conclusion by consensus. Image analysis was performed at both mediastinal (window level, 40 Hounsfield units [HU]; window width, 400 HU) and lung window settings (window level, 700 HU; window width, 1500 HU), using a dedicated workstation. The radiologists were permitted to adjust the window settings if necessary. PPs were defined as variable-size localised pleural thickening of soft tissue, or calcific densities attached along the pleura of the chest wall, diaphragm, and mediastinum on the CTs. The following findings were recorded: number, presence of calcification, maximum width and length, location (chest wall, diaphragm, and mediastinal pleura), and extent score of PPs. The maximum width was measured from the thickest plaque in the subjects and defined as the maximum vertical distance from the parietal pleura to the interface between the plaque and lung. The maximum length was measured in the largest plaque in the subjects and defined as the longest diameter of the plaque in coronal or sagittal 2D multiplanar reconstruction. For the evaluation of plaque location, the chest wall was divided into right and left, ventral (anterior to the mid-axillary line) and dorsal, and upper (upper 1/2 of the thorax) and lower parts. Finally the extent scores were measured in each hemithorax according to the International Classification of HRCT for Occupational and Environmental Respiratory Diseases (ICOERD) classification system [19]. In particular, the involvement of the circumference of the lung, excluding the mediastinum, was calculated by combining maximum lengths of pleural plaques on axial image at the mid-thoracic level as follows: 0 = no plaques; 1 = up to 1/4; 2 = 1/4–1/2; and 3 > 1/2 of the circumference of the chest wall. The total extent score was defined as the sum of the extent scores of the right and left hemithorax (min. 1, max 6). The thickening score was assessed by measuring the thickest plaque of each hemithorax assigning the score as follows: 0 = no plaques; 1 = 1–5 mm; 2 = 5–10 mm; 3 > 10 mm. The total thickening score was defined as the sum of the scores of the right and left hemithorax (min. 1, max 6). ICOERD classification was also used to report parenchymal findings and in particular the presence of well-defined rounded opacities, irregular and/or linear opacities, ground glass opacities, honeycombing, emphysema, and large opacities.

### 2.4. Statistical Analysis

The pleural findings detected by the readers were collected, and the results expressed as mean +/− standard deviation (SD). A descriptive statistical analysis was performed and variables were expressed as percentages. Student's *t*-test for paired samples was used to compare the maximum width of reported and underreported PPs. A *p* value of less than 0.05 was considered to indicate a significant difference. The statistical review of the study was performed by a biomedical statistician. The analysis was performed using Stata version 8.0 (Stata Corp., College Station, Texas).

## 3. Results

**Figure 1:**
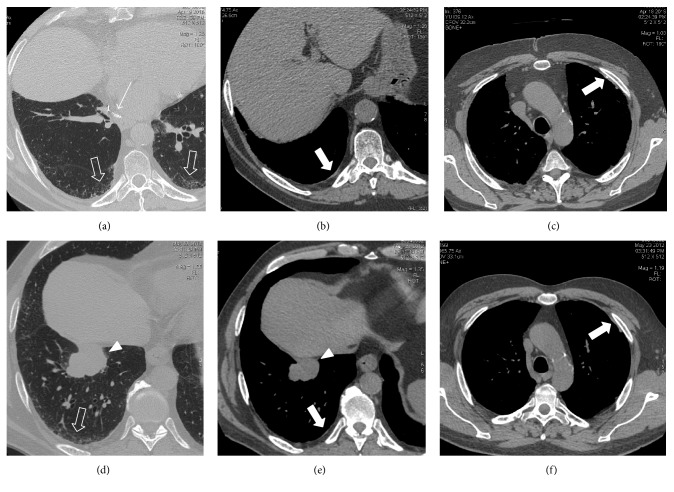
(a–f) Follow-up CT in a 65-year-old man 4 years after lower right lobectomy for lung cancer (arrow in (a)) demonstrates asbestosis (white open arrows in (a)) and bilateral PPs (white solid arrows in (b) and (c)). These CT findings were present since 2012 as showed by the presurgical staging CT (lung cancer, arrowhead in (d) and (e); asbestosis, white open arrows in (d); PPs, white solid arrows in (e) and (f)).

Thirty out of 1512 CTs (2%) examinations were excluded because of motion artefacts (*n* = 10), insufficient image resolution (*n* = 6), or partially explored lung (*n* = 14). The remaining 1482 chest CTs represent the final cohort of the study. PPs were found in 76 out of 1482 patients (5.1%); in thirteen out of 76 (17.1%) CTs were performed because of clinical suspicion of asbestos exposure and 5 of them (38%) were underreported by radiologist. In the remaining 63 cases (82.9%) there was no clinical suspicion of asbestos exposure at the time of CTs (incidental findings). Among these 63 cases, a history of asbestos exposure was established in 53 (84.1%) by recording their work history, analysing clinical reports, and acquiring information from the patients, after our blinded image analysis. In thirty-eight of these 63 patients (60.3%) PPs were not mentioned in the final report of CTs (underreported) (Figure 1). After consensus all the 76 patients with PPs at CTs (56 men, mean age 67 years, range 55–84, and 2 women of 63 and 72 years of age, resp.) were scored by the study reviewers as showing at least one pleural plaque. The jobs features of patients with history of asbestos exposure (66/76, 86.8%) are summarised in Table 1 whereas all PPs features are summarised in Tables 2 and 3, respectively. Among the 66 cases with history of asbestos exposure, 65 had multiple and bilateral PPs whereas 1 had two monolateral PPs. The 10 cases of PPs without a history of occupational asbestos exposure had a single and unilateral plaque in 8 cases and multiple and bilateral plaques in 2 cases. There were less than 5 plaques in 17 cases (22.4%), uncalcified in 23 (30.3%), partially calcified in 38 (50%), and completely calcified in 15 (19.7%). With regard to the distribution on the pleural surface, the chest wall was the most common location (71/76, 93.4%), followed by the diaphragm (43/76, 56.5%) and the mediastinum (12/76, 15.7%). Chest wall PPs had a particular distribution along the craniocaudal and anteroposterior directions: the lower half was more commonly involved than the upper one (26/76, lower half, 34.2%; 11/76, upper half, 14.5%; 34/76, both the regions, 44.73%) and in the upper half there was a slight ventral predominance (24/45, upper ventral, 53.3%; 18/45, upper-dorsal, 40%; 3/45, both the regions, 6.7%), whereas in the lower half there was a clear dorsal predominance (37/60, lower-dorsal, 61.7%; 16/60, lower-ventral, 35.5%; 7/60, both the regions, 2.8%). Diaphragmatic pleurae were bilaterally involved in 21 cases (27.6%), only on the right side in 15 cases (19.7%) and only on the left side in 7 (9.2%). Mediastinal pleura had no cases with bilateral involvement and the left side had more plaques than the right side (9, 11.8%, versus 3, 3.9%). Among the 10 cases of PPs without a history of occupational asbestos exposure, six out of 8 cases with a single plaque were attributable to pleuritis, caused by previous episodes of pneumonia, and the other 2 were probably caused by the hemothorax due to previous trauma. In the remaining 2 cases of bilateral and multiple plaques it is plausible that there was environmental asbestos exposure. PPs mean width of all 76 cases was 5.5 ± 2.96 mm (range 1–12.2) and mean length was 62.9 ± 49.1 mm (range 2–178). According to the ICOERD classification, extent and width scores were as follows:* extent score* (mean): right hemithorax 1.5 ± 0.7; left hemithorax 1.6 ± 0.8; total mean score 3.1 ± 1.5 (range 1–6); width score (mean): right hemitorax 1.4 ± 0.6; left hemitorax 1.3 ± 0.6; total mean score 2.7 ± 1.2 (range 1–6). The other findings resulting from ICOERD classification are summarised in Table 4. Furthermore there was not a significant difference in PPs mean width between reported and underreported PPs (5.4 ± 2.7 mm versus 5.5 ± 3.3 mm, *p* > 0.05).

## 4. Discussion

Incidental findings on radiographic examinations have been available since the beginning of diagnostic radiology. With the introduction of cross-sectional imaging, the detection of such findings became more common, and their recognition was typically believed to be useful by leading to early detection of subclinical disease, and probably to better outcomes [20]. Incidental abnormalities of the pleura are most commonly pleural effusions, followed by focal abnormalities such as noncalcified or calcified PPs. Clinically significant incidental pleural abnormalities, namely, indeterminate pleural masses, were rarely reported among lung cancer screening studies in less than 1% of subjects [21]. Our study highlights that PPs, that are considered to be indicators of asbestos exposure and the most common manifestation of inhalation, retention, and biological effect of asbestos fibres, can be detected, as incidental findings, on chest CTs, even if there is no specific suspicion, and that radiologists tend to underreport them. Underreporting and undercompensation of occupational diseases, especially asbestos-related ones, is a widespread phenomenon in many countries, so that various authors identified the need for action to reduce underestimation and to improve current reporting practices and compensation policies [22]. The explanations for this phenomenon could be found in different reasons. First of all, it is necessary to recognise five main scenarios: (1) the radiologist is not aware of the clinical suspicion of asbestos exposure; (2) the radiologist is aware of the clinical suspicion of asbestos exposure and he is sufficiently familiar with CT findings in occupational diseases; (3) the radiologist is aware of the clinical suspicion of asbestos exposure but his experience in the field of occupational diseases is not sufficient; (4) the radiologist is aware of the patient's job history but is not aware of possible asbestos exposure in that job (e.g., not all radiologists are aware of possible asbestos exposure in plumbers!); (5) the CT technique is not sufficiently adequate to demonstrate PPs. Regarding the first two points, the underreporting of PPs could be due to observer or perceptual errors and in particular to both scanning and alliterative error. In the former (scanning or perceptual error) error is the result the radiologist's failure to fixate on the area of the lesion, in these cases the pleurae. Scanning or perceptual errors, in general, are related to multiple psychophysiological factors, including level of observation alertness, observer fatigue, duration of the observation task, any distracting factors, conspicuity of the abnormality, and many other factors, such as the absence of a specific clinical suspicion when searching PPs in the first clinical scenario [23–26]. In an MDCT examination, the high number of CT images substantially contributes to the perceptual error; however, the reduction in the number of images (i.e., image retroreconstruction with a thicker slice) should be discouraged because of the reduction of the CT diagnostic capabilities [27]. Alliterative error, that could also occur in the third scenario, is a perceptual error that results from the influence a radiology report has over another radiologist. This type of perceptual error occurs because the radiologist reads the old report before looking at the images; if the first radiologist missed it, the next radiologist is likely to miss it as well [28]. In our case history, among the 63 patients with PPs as incidental findings, 43 patients have at least one previous CT, with a negative report for pleural findings and in particular PPs. In the third and fourth clinical scenarios (the radiologist is aware of the clinical suspicion of asbestos exposure but his experience in the field of occupational diseases is not sufficient or the radiologist is aware of the patient's job history but is not aware of possible asbestos exposure in that job) the error could also be attributed to mistaken exam interpretation or cognitive error. A cognitive error is the result of a failure to correctly interpret a perceived radiological abnormality because of insufficient experience or knowledge or an underestimation of one or more signs that would have prompted the correct diagnosis. It is a common condition as occupational diseases are a niche field in radiology and also due to the variety of CT findings in environmental and occupational exposure, although this type of error could be reduced if the correct diagnostic predictions based on clinical information are suggested. In cognitive error the radiologists' awareness of PPs and focal pleural thickening mimicking PPs on chest CTs could also be considered. In fact even if the diagnosis of PPs is commonly straightforward, numerous causes of focal pleural thickening may nevertheless be seen and misinterpreted in routine practice, producing both false positive and false negative results that may lead to medicolegal consequences or can cause underreporting and undercompensation of occupational diseases. Reaching a correct diagnosis of PPs requires a good knowledge of normal locoregional anatomy (transversus thoracic muscle, subcostal muscle, extrapleural fat, etc.), different features of PPs, and common pitfalls in their diagnosis (focal dependent pleural thickening, pseudoplaques in sarcoidosis and silicosis) [18, 29, 30]. Last but not least, in order to reduce underestimation and to improve current reporting practices of PPs, technical approaches in chest CT execution should also be rigorous. Thin-section CT acquisition (≤1.25 mm) in full inspiration is recommended for scanning the thorax, in order to avoid missing tiny, thin, and uncalcified PPs. Furthermore, considering the fact that PPs more commonly involve lower pleura than the upper, the dorsal regions of basal thoracic wall and the diaphragm, and that asbestosis also prefers the dorsal regions of the lower lobes, the patient should be placed in a prone position during CTs. However, if the CTs are performed with the patient in a supine position, the presence of pleural thickening in the dorsal regions, in the absence of PPs in other regions of the pleura, requires an additional acquisition in prone position. This approach will differentiate a real plaque from reversible dependent pleural thickening [29]. According to a recent study by Kim et al. [31], an interesting distribution of PPs was found, in particular:* diaphragmatic plaques* were distributed more commonly on the right side, since the right diaphragmatic dome has a large interface with the lung;* mediastinal plaques* were distributed more commonly on the left side due to anatomical and mechanical factors such as larger interface with the lung and the pulsating left ventricle pushing the left mediastinal pleura against the adjacent left lung with more mechanical stress than the right mediastinal pleura;* chest wall pleural* plaques more commonly involved both the basal sides due to combination of high ventilation and gravity in these lung regions. Inferior pleura is more frequently involved than the upper; basal thoracic wall and diaphragm localisations generally prefer dorsal regions; on the contrary apices of the thoracic cavity show a prevalent ventral distribution. Furthermore, in our case history, PPs mean thickness and extension were, respectively, 5.5 ± 2.96 mm (range 1–12.2) and 62.9 ± 49.1 mm (range 2–178). At these sizes their CT identification should be easy, if the pleura is carefully and systematically analysed on all chest images, even if clinical suspicion of asbestos exposure is not present. This study has some limitations. Firstly the size of this case population may still be not sufficiently comprehensive to fully understand whether and how radiologists report the pleural findings on standard chest CTs. Nevertheless, the observed prevalence of PPs highlights the importance of looking carefully at the pleura, which is more assessable nowadays with the use of thin slice thickness on CTs.

## 5. Conclusions

In conclusion, this study shows that PPs can be detected on CTs even in absence of clinical suspicion of asbestos exposure, but regardless of their potential relevance, they are often underreported. Knowledge of the typical appearance and location of PPs is crucial for their correct recognition and their differentials. However the patient's job history should always be kept in mind and the associated findings carefully looked at.

## Figures and Tables

**Table 1:** Jobs' features of patient with history of asbestos exposure.

*Industrial sector*	
Metal workers	9
Asbestos sheets producers	2
Asbestos insulation removers	7
*Construction sector*	
Bricklayer	21
Plumber	5
Aqueduct technician	3
Boiler technician	3
*Transport sector*	
Shipyard workers	5
Dockers	3
Mechanics	3
*Craftsmanship*	
Shoemaker	2
Glassworkers	3

*Total*	66

**Table 2:** PPs distribution.

Chest wall	
*Upper versus lower*	45 (59.2%) versus 60 (78.9%)
Upper	11 (14.5%)
Lower	26 (34.2%)
Both	34 (44.73%)
*Total*	71 (93.4%)

Upper ventral versus upper dorsal	24/45 (53.3%) versus 18/45 (40%)
Both	3/45 (6.7%)
Lower ventral versus lower dorsal	16/60 (35.5%) versus 37/60 (61.7%)
Both	7/60 (2.8%)

Diaphragm	

Right	15 (19.7%)
Left	7 (9.2%)
Both	21 (27.6%)

*Total*	43 (56.5%)

Mediastinum	

Right	3 (3.9%)
Left	9 (11.8%)
Both	0%

*Total*	12 (15.7%)

**Table 3:** PPs characteristics.

*Number of plaques*	
Less than 5	17 (22.4%)
5 or more	59 (77.6%)

*Calcification*	
Uncalcified	23 (30.3%)
Partially calcified	38 (50%)
Completely calcified	15 (19.7%)

*Involvement of hemithorax*	
Unilateral	9 (11.8%)
Bilateral	67 (88.2%)

*Maximum width*	
Range	1–12.2 mm
Mean	5.5 mm

*Maximum length*	
Range	2–178 mm
Mean	62.9 mm

*Extent score (mean)*	
Right	1.5
Left	1.6

*Total*	3.1

*Width score (mean)*	
Right	1.4
Left	1.3

*Total*	2.7

**Table 4:** Additional lung ICOERD findings.

Lung ICOERD features	Patients	Abnormalities significance (number of cases)
*Normal lung parenchyma*	21 27.6%	

*Well defined rounded opacities*	18 23.7%	(i) Postinflammatory (8) (ii) Silicosis (2) (iii) Metastasis (3) (iv) Sarcoidosis (1) (v) Uncertain significance (4)

*Irregular and/or linear opacities*	20 29.3%	(i) Lung fibrosis with UIP consistent pattern (2) (ii) Organizing pneumonia (1) (iii) Hypersensibility pneumonia (1) (iv) Sarcoidosis (1) (v) Asbestosis (6) (vi) Pulmonary infarction (2) (vii) Uncertain significance (7)

*Ground glass opacities*	8 10.5%	(i) Lung cancer (3 cases) (ii) Desquamative interstitial pneumonia (2) (iii) Uncertain significance (3)

*Honeycombing*	4 5.2%	(i) Lung fibrosis with UIP consistent pattern (3) (ii) Hypersensibility pneumonia (1)

*Emphysema*	16 21%	

*Large opacities*	5 6.8%	(i) Lung cancer (2 cases) (ii) Rounded atelectasis (1) (iii) Mesothelioma (1) (iv) Hamartoma (1)
